# Clinical Profiles, Survival, and Lung Function Outcomes in ANCA-Associated Interstitial Lung Disease: An Observational Study

**DOI:** 10.3390/jcm14010229

**Published:** 2025-01-03

**Authors:** Cristina Valero-Martínez, Claudia Valenzuela, Juan Pablo Baldivieso Achá, Elisa Martínez-Besteiro, Patricia Quiroga-Colina, Arantzazu Alfranca, Esther F. Vicente-Rabaneda, Susana Hernández Muñiz, Santos Castañeda, Rosario García-Vicuña

**Affiliations:** 1 Rheumatology Department, Hospital Universitario de La Princesa, IIS-Princesa, 28006 Madrid, Spain; cristina.valmart@gmail.com (C.V.-M.); juanpablobaldiviesoacha@gmail.com (J.P.B.A.); pquiroga@alumni.unav.es (P.Q.-C.); efvicenter@gmail.com (E.F.V.-R.); scastas@gmail.com (S.C.); 2 ILD Unit, Pulmonology Department, Hospital Universitario de La Princesa, IIS-Princesa, 28006 Madrid, Spain; claudiavale@hotmail.com (C.V.); elisa.martinez.besteiro@gmail.com (E.M.-B.); 3 Immunology Unit, Hospital Universitario de La Princesa, IIS-Princesa, 28006 Madrid, Spain; mariaaranzazu.alfranca@salud.madrid.org; 4 Medicine Department, School of Medicine, Universidad Autónoma of Madrid, 28049 Madrid, Spain; 5 Radiology Unit, Hospital Universitario de La Princesa, IIS-Princesa, 28006 Madrid, Spain; susanitahemu@yahoo.es

**Keywords:** anti-neutrophil cytoplasmic antibodies, ANCA, interstitial lung disease, ILD, vasculitis, AAV

## Abstract

**Background/Objectives**: Anti-neutrophil cytoplasmic antibodies (ANCAs) have been found in interstitial lung disease (ILD) in recent years, although its impact on ILD prognosis is less known. To date, ANCAs are not included in the interstitial pneumonia with autoimmune features (IPAF) definition criteria. Therefore, ANCA-ILD, in the absence of known ANCA-associated vasculitis (AAV), could be underdiagnosed. Our aim was to analyze the clinical profile and prognosis of ANCA-ILD patients. **Methods**: Patients diagnosed with ILD and positive ANCA were enrolled in a retrospective, monocentric cohort study. Lung function outcomes and mortality were assessed according to clinical, serological, radiological, and treatment characteristics. Survival was analyzed using Kaplan–Meier curves and Cox regression models. **Results**: A total of 23 patients were included, mostly women, with a median time from ILD diagnosis of 36 (24–68) months and a predominant anti-MPO pattern (56.5%). Nearly half of the patients had AAV, mostly microscopic polyangiitis (MPA). The presence of AAV was significantly associated with anti-MPO antibodies and an NSIP radiographic pattern. Overall, the fibrotic pattern (either UIP or fibrotic NSIP) was the most common (73.9%), mainly UIP (51.2%). However, it appeared less frequently in the AAV-ILD group. During follow-up, lung function impairment or radiological progression was observed in 65.2% of patients. Cumulative mortality incidence was high (43.4%), largely due to ILD itself (80%). A UIP pattern was associated with a higher and earlier mortality (HR 34.4 [1.36–132]), while the use of immunosuppressants showed a trend towards lower ILD-related death. **Conclusions**: In our cohort, ANCA-ILD patients mostly presented with fibrotic patterns, with AAV in almost half of the cases and a high and early mortality rate, which suggests the need to assess ANCA in all ILD patients.

## 1. Introduction

Anti-neutrophil cytoplasmic antibodies (ANCAs) are autoantibodies that target antigens in the cytoplasmic granules of neutrophils and the lysosomes of monocytes. The antigens proteinase-3 (PR3) and myeloperoxidase (MPO) are found in neutrophil primary granules and represent the main targets of ANCA [1]. Two classic immunofluorescence ANCA patterns have been described: (i) cytoplasmic staining with marked interlobular accentuation (C-ANCA) mainly associated with the antigen PR3, and (ii) perinuclear staining with nuclear extension (P-ANCA), frequently associated with the antigen MPO. The detection of ANCA associated with the diagnosis of small-vessel vasculitis has changed the definition and nomenclature of the entity, now called ANCA-associated vasculitis (AAV) [2]. ANCA positivity is highly specific and has a high predictive value in the clinical setting of patients suspected of having vasculitis [1].

The relationship between interstitial lung disease (ILD) and ANCA or AAV has been observed in recent years, and ILD usually precedes the diagnosis of AAV [3,4]. However, it is still uncertain whether lung fibrosis is a definitive manifestation of AAV [3]. Large cohorts of patients with a diagnosis of idiopathic pulmonary fibrosis (IPF) have reported ANCA-positive prevalence rates ranging from 9.8 to 17%, mostly anti-MPO antibodies, and especially in the setting of associated AAV [5,6,7]. It is important to note that ANCA can become positive during the course of ILD, with a reported ANCA seroconversion rate in around 2% patients per year [6,8]. Prevalence rates of ILD in patients with AAV are 23% in granulomatosis with polyangiitis (GPA) and 45% in microscopic polyangiitis (MPA) [4,9].

Patients with ILD who are ANCA-positive but do not have vasculitis (isolated ANCA-ILD) have been shown to have a higher frequency of fibrotic ILD, with the usual interstitial pneumonia (UIP) pattern observed in up to 78% of cases [3,4,9]. However, whether ANCA positivity or a given specificity implies a worse prognosis in isolated ANCA-ILD patients remains unclear. In this regard, some data show an excess of premature AAV mortality related to respiratory disease in anti-MPO positive patients, particularly higher in MPA patients with pulmonary fibrosis [3,9,10]. Unfortunately, ANCA detection is not routinely performed in patients with newly detected ILD. In IPF guidelines, serological testing to rule out connective tissue disease is recommended for patients with ILD of apparently unknown cause with suspected IPF, with ANCA testing added only if vasculitis is suspected [11]. To date, ANCA determination has not been included in the serological domain criteria for interstitial pneumonia with autoimmune features (IPAFs) [12]. Therefore, ANCA-ILD in the absence of well-recognized findings of vasculitis or isolated ANCA-ILD could be underdiagnosed.

There is no specific therapeutic approach for isolated ANCA-ILD, and the effectiveness of glucocorticoids (GCs) or immunosuppressive (IS) agents in this context remains uncertain. This study aims to analyze clinical, imaging, and therapeutic features, as well as long-term outcomes in patients with ANCA-associated ILD with or without AAV in our center.

## 2. Materials and Methods

### 2.1. Patients and Study Design

This was a single-center retrospective cohort study conducted in a third-level center in Madrid, Spain. We retrieved all ANCA assessments performed by the immunology department between January 2011 and March 2024. Patients diagnosed with ILD and with a minimum follow-up of one-year were selected if they had ≥2 positive ANCA results assessed at least one month apart.

Samples were analyzed for ANCA presence through immunofluorescence on both ethanol- and formalin-fixed neutrophils (Granulocyte Mosaic 13 kit; Euroimmun, Germany), following manufacturer instructions. Those sera positive for either c- or p-ANCA pattern (>1/20 dilution) were subsequently analyzed for anti-PR3 and anti-MPO antibodies by chemiluminescence (Quanta Flash MPO and Quanta Flash PR3 kits; Inova Diagnostics, Spain), following manufacturer instructions. Anti-PR3 and anti-MPO values >20 arbitrary units were considered positive.

Demographic, clinical, serological, and radiological characteristics, pulmonary function tests (PFTs), and therapeutic variables were collected from electronic medical records. PFT parameters, including forced vital capacity (FVC), percentage of predicted value (ppv), and diffusion capacity of the lung for carbon monoxide (DLCO) determined by the single-breath method were collected. Abnormal PFTs were defined by an FVC < 80% ppv or DLCO < 80% ppv, and hypoxemia by oxygen saturation level < 95% or arterial partial pressure of oxygen < 60 mmHg. The ILD pattern in the chest high-resolution computed tomography (HRCT) scans was reported by one thoracic radiologist with at least 8 years of expertise in lecturing thoracic X-rays. All patients with ILD were diagnosed and followed up in the ILD unit by dedicated pneumologists.

### 2.2. Survival and Functional Outcomes

The presence of AAV, abnormal PFTs, hypoxemia, and ILD imaging patterns were analyzed either at baseline or during the follow-up. The main outcomes included survival, functional deterioration from baseline (defined as a decline in FVC ≥ 10% or a decline in FVC around 5–10% and in DLCO ≥ 15% at 12 months), or development of hypoxemia. The functional outcomes were assessed at 6, 12, and 24 months from diagnosis. Hypoxemia and progression of ILD were also reported along the follow-up, at different time-points when HRCTs were available. Survival outcomes were summarized as the death rate (percentage) and incidence rate (IR) of death per 100 patient-years (PY) of follow-up from the time of ILD diagnosis.

### 2.3. Statistical Analysis

Data are expressed as the mean and standard deviation or median and interquartile range (IQR) for continuous variables and counts and percentages (%) for categorical variables. For two-group comparisons of binary data, the Chi-square test or Fisher’s exact test were used. We applied the *t*-test or the Mann–Whitney U test, as appropriate, to compare continuous variables between two groups. The normal distribution of variables was checked using the Shapiro–Wilk test. Bivariate analyses were performed to assess potential factors associated with the presence of AAV, abnormal PFTs, hypoxemia, ILD imaging pattern, either at baseline or in the follow-up, and imaging progression or death. Those factors included sex, age, ILD duration, smoking status, obesity, ANCA specificity, presence of vasculitis, C-reactive protein levels (mg/dL), radiological features or patterns, and the use of GC or IS. The paired samples *t*-test was used to evaluate the evolution between FVC and DLCO during follow-up. A multivariate logistic regression was conducted to predict factors associated with the decline in baseline PFTs, using the covariates significantly associated with the event (*p* < 0.05) in the bivariate analysis. Kaplan–Meier curves were used to represent survival and the log-rank test was used to compare survivor function between patients with or without AAV. Statistical significance was defined as a *p*-value < 0.05. Data analysis was performed using the STATA 14 package (Stata Corp LP, College Station, TX, USA).

## 3. Results

### 3.1. Baseline Clinical Characteristics of the ILD-ANCA Population

Twenty-three patients diagnosed with ILD and positive ANCA results were included (Table 1). Sixteen were women, with a mean age of 70.5 ± 9 years, and 56.5% were active or former smokers, a proportion even higher in the isolated ANCA-ILD group. The median time since ILD diagnosis was 36 (IQR 24–68) months, and anti-MPO was the predominant ANCA specificity (56.5%) in both isolated ANCA-ILD and AAV-ILD patients. A rheumatoid factor was present in six of the patients, but only one was positive for anti-cyclic citrullinated peptide antibodies. Nonetheless, none of the patients were diagnosed with rheumatoid arthritis or connective tissue disease apart from AAV. No positive autoimmunity results were found for other ILD-related autoantibodies such as anti-Jo1, anti-Slc70, or anti-MDA5.

Ten patients (43.4%) were diagnosed with AAV, with half of them being MPA. ILD was diagnosed simultaneously with AAV in 50% of them, and only three patients developed AAV after ILD, with a median time since ILD diagnosis of 20.5 (IQR 19.5–20.5) months. In the bivariate analysis, the likelihood of AAV occurrence was higher in patients with anti-MPO (*p* = 0.046) or a non-specific interstitial pneumonia (NSIP) pattern (*p* = 0.022), but no significant differences in other baseline characteristics were found. The most common AAV manifestation was otorhinolaryngological in four patients, followed by musculoskeletal (3 patients) and renal involvement (3 patients), but only 1/3 subjects presented proteinuria >500 mg/24 h. This case was consistent with rapidly progressive glomerulonephritis and underwent renal biopsy that showed focal extracapillary proliferative glomerulonephritis with negative immunofluorescence staining. Two patients presented with palpable purpura and two presented with peripheral nerve disease. Only two patients presented organ-threatening disease: one with cardiomyopathy and the formerly described patient with rapidly progressive crescentic glomerulonephritis. None of them presented with diffuse alveolar hemorrhage (DAH).

### 3.2. Radiological and Lung Function Findings

**Figure 1:**
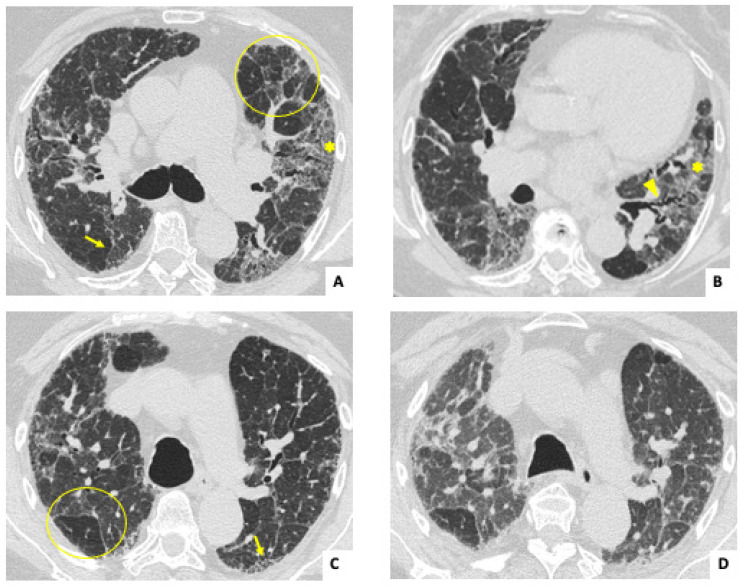
Inspiratory axial HRCT images (**A**–**C**) in a 65-year-old woman diagnosed with isolated ANCA-ILD and positive C-ANCA. Pictures show reticulation (arrows), traction bronchiectasis (arrowhead), and ground glass opacities (asterisks), with no craniocaudal gradient. Bilateral mosaic pattern (geographic regions of decreased lung density) can also be detected (circles). Expiratory CT (**D**) confirms the presence of air trapping, and the mosaic pattern is highlighted compared to C. Findings suggest a non-UIP fibrotic interstitial lung disease.

**Figure 2:**
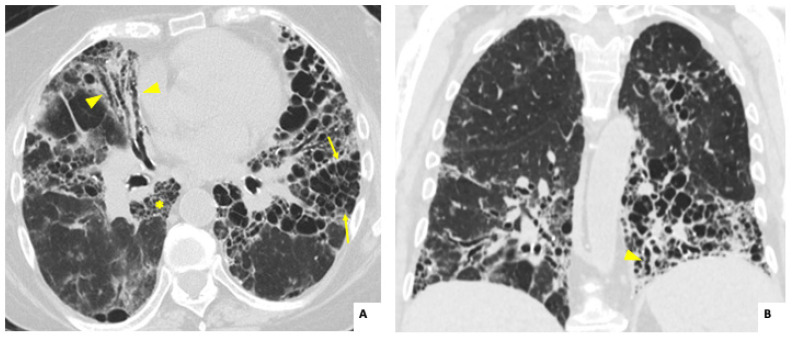
Axial (**A**) and coronal (**B**) high-resolution computed tomography images showing extensive fibrotic interstitial lung disease in a 70-year-old woman, diagnosed with isolated ANCA-ILD and anti-MPO positivity. Note the basilar predominance of honeycombing (arrows), traction bronchiectasis (arrowheads), and reticulation (asterisk); all findings are consistent with the usual interstitial pneumonia (UIP) pattern.

The most frequent radiological findings (Table 1) were traction bronchiectasis, ground-glass opacities, and reticulation in both ANCA-ILD and AAV-ILD groups. Overall, the main radiological subtype was a fibrotic pattern (either UIP or fibrotic NSIP) in 17 patients (73.9%) (Figure 1 and Figure 2). However, non-fibrotic patterns (cellular NSIP and organizing pneumonia [OP]) were more frequent in the AAV-ILD group (60%) compared to patients with isolated ANCA-ILD (30.7%).

At diagnosis, 70% of patients had abnormal PFTs, being numerically higher in the ANCA-ILD vs. AAV-ILD group (76.9% vs. 40%), whereas only 21.7% had hypoxemia at baseline. In the multivariable logistic regression model, the presence of male sex, smoking history, anti-MPO positivity, or the presence of a radiological fibrotic pattern was associated with reduced PFTs at diagnosis, but only the correlation with radiological fibrotic pattern was statistically significant (*p* = 0.025). PFTs could not be performed in three patients at the time of diagnosis.

### 3.3. Treatment Administered

In most of the patients, treatment for ILD included GC (n = 19; 82.6%) followed by IS agents (n = 15; 65.2%), with mycophenolate (n = 6, 26%) and rituximab (n = 5, 21.7%) as the most prescribed drugs. All patients with AAV-ILD received both IS and GC. Cyclophosphamide was prescribed in three patients with AAV-ILD, two of them due to rapidly progressive lung disease and the other one due to rapidly progressive crescentic glomerulonephritis. Three patients with eosinophilic granulomatosis with polyangiitis received mepolizumab.

Among patients with isolated ANCA-ILD, 38.4% (n = 5/13) received IS and 69.2% (n = 9/13) received GC. Antifibrotics were only used in four cases, and around one-third of the patients required supplementary long-term oxygen therapy (LTOT). In four cases of isolated ANCA-ILD, no ILD specific treatment was provided, due to the absence of symptoms or normal PFTs.

### 3.4. Pulmonary Function Outcomes During Follow-Up

The median follow-up period of ILD from diagnosis in the global population was 36 months (IQR, 24–38) without significant differences between patients with isolated ANCA-ILD and patients with associated vasculitis (Table 2). During 85 patient-years (PY) of follow-up, more than half of the patients experienced a decline in PFTs from baseline (65.2%), hypoxemia (52.2%), or showed radiological progression in HRCT (65.2%) at 24 months (Table 2). In the AAV-ILD group, more patients experienced hypoxemia or radiological progression compared to the isolated ANCA-ILD group, but no statistically significant differences were reached (*p* = 0.673 and *p* = 0.680, respectively).

Notably, about one-third of patients (34.8%) presented with rapidly progressive ILD (RP-ILD) during follow-up, mainly in the AAV-ILD patients (50% vs. 23% isolated ANCA-ILD group), but this trend was not statistically significant (*p* = 0.221). After 12 months, the median % DLCO significantly decreased in both groups similarly, while the FVC remained stable (Table 2). After 24 months, PFTs remained stable for those whose data were available (n = 14).

In the bivariate analysis, factors associated with functional impairment during follow-up were a higher mean age (*p* = 0.048), abnormal PFTs at diagnosis (*p* = 0.05), the lowest baseline levels of FVC% (*p* = 0.0004), a radiological fibrotic pattern (*p* = 0.05), and a greater progression on HRCT during follow-up (*p* = 0.006). Male sex or anti-MPO was also higher in patients with a decline in PFTs but no statistically significant differences were found (*p* = 0.345 and *p* = 0.179, respectively).

### 3.5. Survival and Risk Factors

Over 85 PY of follow-up from diagnosis, 10 patients died (43.4%), largely attributed to the ILD itself (n = 8/10; 80%), with a similar frequency between patients with or without AAV. The overall incident mortality rate was 11.7 per 100 PY (95% CI, 6.3–22), being higher in males compared with females (23.9 vs. 8.8 per 100 PY), but this difference did not reach statistical significance (*p* = 0.07). No significant difference was found by age older than 65 years (11.1 > 65 years vs. 13.8 < 65 years per 100 PY; *p* = 0.36) or by the presence of AAV (11.9 AAV vs. 11.5 isolated ANCA-ILD per 100 PY; *p* = 0.49).

In the bivariate analysis, death attributed to ILD was influenced by the presence of HRCT honeycombing areas (*p* = 0.006), RP-ILD (*p* = 0.006), a decline in PFTs (*p* = 0.019), hypoxemia (*p* = 0.001), or radiological progression in HRCT (*p* = 0.019) during follow-up. However, death induced by ILD was not related to sex (*p* = 0.657) or higher age (*p* = 0.13). The use of IS was associated with less mortality induced by ILD in both AAV-ILD and isolated ANCA-ILD groups, but this difference did not reach statistical significance (*p* = 0.442 and *p* = 0.794, respectively).

**Figure 3:**
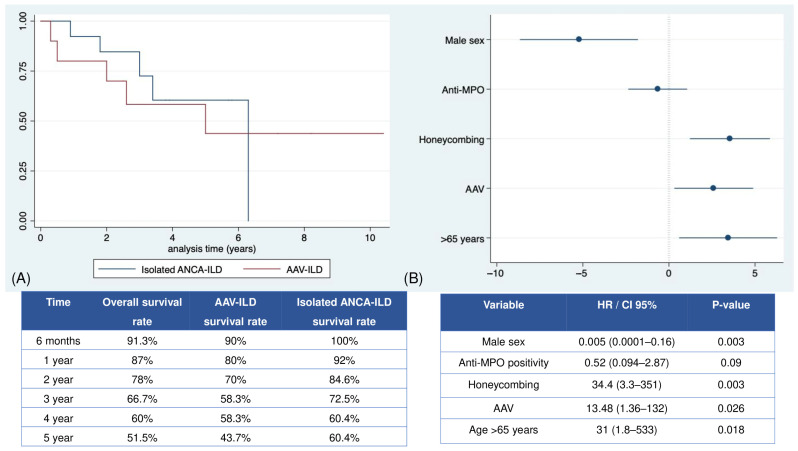
Overall and stratified survival in the population with ILD and positive ANCA. (**A**) Kaplan–Meier survival curves for isolated ANCA-ILD or AAV_ILD based on the cumulative probability of survival. (**B**) Forest plot of the regression Cox analysis of the risk factors associated with premature mortality. Abbreviations: ANCA: anti-neutrophil cytoplasmic antibody; AAV: ANCA-associated vasculitis; Anti-MPO: anti-myeloperoxidase antibody; CI: confidence interval; HR: hazard ratio. ILD: interstitial lung disease.

In the Kaplan–Meier overall survival analysis, the cumulative 3-year survival rate was 66.8% (95% CI, 64–95%) and half of the deaths (n = 5/10) occurred within two years of follow-up (Figure 3A). No statistically significant differences in survivor functions by the log-rank test were observed between AAV-ILD and isolated ANCA-ILD (*p* = 0.81) in all-cause mortality. In the multivariate Cox regression analysis (Figure 3B), the presence of honeycombing areas, AAV, and age >65 years was significantly associated with premature death.

## 4. Discussion

The main findings of our study can be summarized as follows: (1) in patients with ILD with positive ANCA, nearly half of the patients have a simultaneous or subsequent diagnosis of AAV, mostly MPA; (2) cumulative global mortality over a median of 3 years of follow-up was high, mainly due to the ILD itself; (3) premature death was associated with the presence of a UIP pattern on HRCT, age older than 65 years, and AAV. Those results reinforce the need for an early diagnosis of isolated ANCA-ILD, due to prognostic and therapeutic implications. In this regard, while many retrospective cohorts have explored the link between AAV and ILD, there is limited information available on the characteristics and prognosis of ANCA-positive patients with isolated ILD.

The correlation between ILD and AAV diagnosis has been explored in multiple retrospective AAV cohorts, and the increased prevalence of MPA, P-ANCA, and anti-MPO-positive antibodies is well established [5,7,13,14,15,16,17,18,19,20,21,22,23]. The estimated prevalence of AAV in ANCA-positive-IPF patients ranges between 30 and 50%, with MPA predominance [5,7,19,21,22,23,24,25]. Our results confirmed these data and showed that AAV prevalence was 43.4% in our ANCA-ILD patients, with half of them being MPA, and with a preponderance for anti-MPO specificity. Also consistent with our findings, AAV development has been described in the first two years of ILD follow-up [7,13], after or concurrently with ILD diagnosis [7,9,10,14,16,20,24]. The risk factors that have been classically associated with AAV development in patients with preexisting ILD include elevated inflammatory markers, anemia, radiological UIP pattern, older age, fever, anti-MPO positivity, or higher serum ANCA levels [5,24,25,26,27,28,29]. In our series, the presence of concurrent AAV in ANCA-ILD was associated with anti-MPO antibodies or radiological NSIP patterns, but we could not identify other additional factors.

There is no clear differential profile in vasculitic manifestations between AAV patients with or without ILD. Various studies indicate that patients with AAV-ILD generally have less severe systemic symptoms [10,14,15,22,23], which aligns with our findings, where the incidence of life-threatening disease was low. Some reports have found fewer ear, nose, and throat symptoms in AAV-ILD patients compared to those with non-ILD AAV [10,23]. In contrast, otorhinolaryngological manifestations were the most common in our patients with AAV-ILD, though we could not perform a comparison with non-ILD AAV cases.

The radiological presentation of AAV-ILD vs. isolated ANCA-ILD or IPF seems to be indistinguishable [4,8,25]. Similar findings had been reported in lung biopsies, which did not reveal histopathological evidence of capillaritis or vasculitis that could guide the definite diagnosis [4]. Regarding IPF, some studies reported that ANCA-positive patients were more likely to exhibit ground-glass opacities and honeycombing compared to those with negative ANCA [25,28]. The main radiological pattern observed in both AAV-ILD and isolated ANCA-ILD is UIP [9,13,14,15,16,17,18,19,24], which has also been linked to an increased frequency of MPA-ILD and anti-MPO antibodies [26,30]. Indeed, anti-MPO has shown a direct contribution to lung fibrosis in ANCA-ILD through oxidative stress [31] and the release of proteolytic enzymes or neutrophil extracellular traps (NETs) that trigger fibroblast proliferation and the deposition of extracellular matrix in the lung tissue [4]. In addition, genetic variants associated with a strong susceptibility to IPF have been identified in AAV-ILD [4]. This gathered evidence suggests possible shared pathogenetic mechanisms between both fibrotic ILDs [4].

In our series, the radiological UIP pattern was the most common among all ANCA-ILD patients, showing a higher prevalence in non-AAV patients and without a predominance of anti-MPO specificity. In line with our results, Sakamoto et al. also reported a higher proportion of UIP patterns for isolated ANCA-ILD compared with MPA-ILD patients [27]. Moreover, we identified NSIP as the most common imaging pattern in AAV-ILD, consistent with findings from other studies [8,20].

The presence of ILD has been extensively associated with worse outcomes in AAV [15,18] including a significantly higher risk of death in patients with AAV-ILD vs. non-ILD AAV [13,18,19,23]. In fact, Villeneuve et al. found that ILD had a progressive course in HRCT in half of the AAV-ILD cases [17], and our findings showed that this progression is rapidly progressive in 50% of the AAV patients. Factors linked to a worse prognosis or mortality in AAV-ILD have included the presence of a radiological UIP pattern and a higher lobe fibrosis score, age over 65 years, history of smoking, anti-MPO positivity, progressive course, respiratory failure, ILD exacerbation, requirement of LTOT, and associated DAH and renal involvement [7,8,9,10,13,14,15,16,17,18,21,24,26,27,29,32,33,34]. The overall cumulative mortality rate in AAV-ILD patients is estimated to be one-third [15,34], with reported 5-year survival rates around 66–74% [14,18] and respiratory failure or infections as the leading causes of death [9,17,34]. In our cases, respiratory failure due to ILD was the primary cause of death irrespective of the presence of AAV.

In this regard, the comparative prognosis of isolated ANCA-ILD and AAV-ILD remains unclear due to conflicting results in the literature. Some reported findings suggest that patients with isolated ANCA-ILD also have a poor prognosis or a progressive course [7,8,18,26,27], with related factors overlapping those previously described in AAV-ILD patients [6,24,26,27,29], in line with our results, along with the addition of elevated inflammation markers [4,5,6,7,8,9,29], anti-MPO positivity [7], or high ANCA titers (>50 EU) [28] in some cohorts. In our study, multivariate Cox regression analysis identified UIP pattern, age over 65 years, and AAV as significantly associated with premature death. Concerning survival, several studies found no significant difference in survival rates of patients with MPA-ILD vs. isolated ANCA-ILD [7,18,23]. Takakuwa et al. [18] reported 5-year survival rates of 74% for MPA-ILD vs. 75% for isolated ANCA-ILD, respectively, while Yamakawa et al. [30] found a significantly higher 5-year survival rate in the MPA group (44.6% vs. 30.7% isolated ANCA-ILD). By contrast, growing evidence reveals a significantly lower survival in MPA-ILD vs. isolated ANCA-ILD [8,24,26], which has been linked to an excess of premature mortality related to lung disease in AAV [3,7,9,10]. Those controversial findings are also shown in our series, with similar mortality rates among AAV-ILD vs. isolated ANCA-ILD, but the time to death was longer in the isolated ANCA-ILD group. Given the prognostic significance of ANCA positivity (especially anti-MPO), clinicians managing ILD should routinely assess for ANCA and their specificities in all ILD patients.

Wide uncertainty remains about the impact of GC, IS, or antifibrotics in the treatment of isolated ANCA-ILD. While some reports have suggested that GC may help reduce the progression to AAV and improve clinical and functional outcomes, there is no clear effect on overall survival compared to untreated patients [13,26,28,34]. Likewise, direct research on the effects of IS or antifibrotics in isolated ANCA-ILD patients is lacking. Limited case series in AAV-ILD patients have shown better survival rates in those treated with IS plus GC, rather than GC alone [14,16], but this benefit was not observed in a study with isolated ANCA-ILD patients [35]. Notably, in our sample, either ANCA-ILD or AAV-ILD, the addition of IS to GC in up to half of the population was linked to reduced ILD-related mortality. It is conceivable that the presence of ANCA might have led to the more intensive treatment of ILD, though we did not compare these findings with non-ANCA ILD patients. Finally, a recent review summarizes the limited information about antifibrotic agents in isolated ANCA-ILD and discusses the opportunity of a wise selection of these drugs for widening the treatment options [36].

Our study has several limitations. First, its retrospective design and being conducted at a single center potentially introduced selection bias. Second, a limited sample size complicated stratified analysis and precluded robust conclusions from multivariable models. One difficulty for the inclusion of patients was that many of the identified subjects had only 1 positive ANCA test available, without subsequent determination, so they did not fulfill the entry criterion of 2 ANCA positive tests one month apart, and therefore, those patients could not be included in this study. Third, some missing data, including unavailable PFT results for some patients, should be considered when interpreting the results of the multivariable analysis of prognosis risk factors. Finally, the heterogeneity in drug management and follow-up periods precludes drawing clear conclusions about survival and treatment effectiveness.

## 5. Conclusions

In conclusion, in our study, ANCA-ILD occurs in the context of AAV in almost half of the population, with a predominant fibrotic pattern on HRCT and a high and premature mortality rate both in patients with or without AAV. Given the high prevalence of ANCA in ILD, along with pathogenetic and prognostic implications, those patients with ANCA-ILD should be monitored for the development of AAV and periodically screened for the presence of ANCA specificities.

## Figures and Tables

**Table 1:** Baseline characteristics of patients with ILD and positive ANCA.

Baseline Characteristics	All Patients (n = 23)	Isolated ANCA-ILD (n = 13)	AAV-ILD (n = 10; 5 MPA, 3 GPA, 2 EGPA)	*p*-Value
Sex, n (%)				
Female, n (%)	16 (69.6)	8 (61.5)	8 (80)	0.96
Males, n (%)	7 (30.4)	5 (38.4)	2 (30)	0.40
Smoker, n (%)	3 (13)	2 (15.3)	1 (10)	0.068
Former smoker, n (%)	10 (43.5)	4 (30.7)	6 (60)	0.76
Obesity (BMI > 30), n (%)	5 (21.7)	4 (30.7)	1 (10)	0.339
Age at diagnosis, mean ± SD	70.5 ± 9	70.7 ± 10.7	71.2 ± 6.9	0.38
Presence of other lung diseases				
Asthma, n (%)	3 (13)	1 (7.7)	2 (20)	0.068
COPD, n (%)	6 (26)	5 (38.5)	1 (10)	0.05
OSA, n (%)	2 (8.6)	2 (15.4)	0 (0)	0.092
ANCA pattern				
P-ANCA, n (%)	10 (43.5)	4 (30.7)	6 (60)	0.22
C-ANCA, n (%)	13 (56.5)	9 (69.2)	4 (40)	0.22
Anti-MPO, n (%)	13 (56.5)	5 (38.4)	8 (80)	0.046
Anti-PR3, n (%)	4 (17.4)	3 (23)	1 (10)	0.924
Other autoantibodies				
Rheumatoid factor, n (%)	6 (26)	5 (38.4)	1 (10)	0.179
Anti-CCP, n (%)	1 (4.3)	1 (7.7)	0 (0)	0.06
ANA (>1/160), n (%)	8 (34.7)	6 (46.1)	2 (20)	0.05
Anti-ENA, n (%)	4 (17.4)	4 (30.7)	0 (0)	0.05
Respiratory manifestations (dyspnea or cough), n (%)	15 (65.2)	7 (53.8)	8 (80)	0.673
PFTs values				
% FVC, mean ± SD (n = 20; 9 AAV)	83.5 ± 26	81 ± 27	87 ± 26	0.68
% FEV1, median ± SD (n = 20; 9 AAV)	85.2 ± 26	80 ± 25	92 ± 29	0.16
% DLCO, median ± SD (n = 17; 9 AAV)	66.1 ± 19	66 ± 21	65 ± 17	0.44
Hypoxemia *, n (%)	5 (21.7)	3 (23)	2 (20)	0.85
Abnormal PFT **, (n = 20; 9 AAV)	14 (70)	10 (76.9)	4 (40)	0.161
Radiological features in HRCT				
Nodules, n (%)	2 (8.7)	2 (15.3)	0 (0)	0.092
Lung consolidation, n (%)	8 (34.8)	2 (15.3)	6 (60)	0.039
Ground glass opacities, n (%)	17 (73.9)	9 (69.2)	8 (80)	0.66
Traction bronchiectasis, n (%)	19 (82.6)	13 (100)	6 (60)	0.024
Reticulation, n (%)	15 (65.2)	10 (76.9)	5 (50)	0.179
Honeycomb, n (%)	8 (34.8)	5 (38.4)	3 (30)	0.673
Radiological pattern in HRCT				
UIP, n (%)	12 (52.2)	9 (69.2)	3 (30)	0.161
NSIP, n (%)	6 (26)	1 (7.7)	5 (50)	0.022
Fibrotic NSIP, n (%)	5 (21.7)	4 (30.7)	1 (10)	0.09
OP, n (%)	5 (21.7)	3 (23)	2 (20)	0.673
C-reactive protein, mean ± SD	2.3 ± 3.2	0.48 ± 0.3	4.8 ± 3.6	0.001

Abbreviations: AAV: ANCA-associated vasculitis; ANA: antinuclear antibody; ANCA: anti-neutrophil cytoplasmic antibody; Anti-CCP: anti-cyclic citrullinated peptide antibody; Anti-MPO: anti-myeloperoxidase antibody; Anti-PR3: anti-proteinase 3 antibody; BMI: body mass index; COPD: chronic obstructive pulmonary disease; DLCO: diffusion capacity of carbon monoxide; EGPA: eosinophilic granulomatosis with polyangiitis; ENA: extractable nuclear antigen; FEV1: forced expiratory volume in the first second; FVC: forced vital capacity; GPA: granulomatosis with polyangiitis; HRCT: high-resolution computed tomography; ILD: interstitial lung disease; MPA: microscopic polyangiitis; NSIP: non-specific interstitial pneumonia; OP: organizing pneumonia; OSA: obstructive sleep apnea; PFTs: pulmonary function tests; SD: standard deviation; UIP: usual interstitial pneumonia. * Hypoxemia is defined as oxygen saturation level (SaO_2_) < 95% or arterial partial pressure of oxygen (PaO_2_) < 60 mmHg. ** Abnormal PFT is defined as FEV1 < 80%, FVC < 80%, or DLCO < 70%.

**Table 2:** Outcomes in patients with ILD and positive ANCA.

Variables Analyzed During Follow-Up	All Patients (n = 23 Patients)	Isolated ANCA-ILD (n = 13 Patients)	AAV-ILD (n = 10 Patients: 5 MPA, 3 GPA, 2 EGPA)	*p*-Value
ILD follow-up period (months), median (IQR)	36 (24–68)	36 (24–47)	35.5 (24–86)	0.77
Deterioration of PFT during follow up *, n (%)	15 (65.2)	8 (61.5)	7 (70)	0.673
%FVC, median (IQR)				
6 months (n = 16; 8 AAV)	80.5 (73.5–100.5)	90 (77–100.5)	75 (67.5–104)	0.115
12 months (n = 17; 8 AAV)	75 (68–106)	78 (73–106)	69 (64–104)	0.027
24 months (n = 14; 5 AAV)	84 (69–102)	81 (61–102)	87 (71–102)	0.43
%DLCO, median (IQR)				
6 months (n = 14; 7 AAV)	72.6 (46–81)	72 (52.6–93)	73.2 (45.9–79)	0.554
12 months (n = 14; 7 AAV)	45.5 (30–67)	47 (44–83)	44 (29–53)	0.712
24 months (n = 11; 3 AAV)	47 (35–73)	47 (36–75)	69 (26–73)	0.076
Hypoxemia during follow-up **	12 (52.2)	7 (53.8)	5 (50)	0.855
Radiological progression in HRCT, n (%)	15 (65.2)	9 (69.2)	6 (60)	0.685
RP-ILD, n (%)	8 (34.8)	3 (23)	5 (50)	0.673
Death rate, n (%)	10 (43.5)	5 (38.4)	5 (50)	0.58
Incidence mortality rate ^&^ (95% CI)	11.7 (6.3–22)	11.5 (4.8–27)	11.9 (4.9–28)	0.48
Cause of death				
ILD, n (%)	8 (34.8)	5 (38.4)	3 (30)	0.673
Infection, n (%)	1 (4.3)	0 (0)	1 (10)	0.06

Abbreviations: AAV: ANCA-associated vasculitis; ANCA: anti-neutrophil cytoplasmic antibody; CI: confidence interval; DLCO: diffusion capacity of carbon monoxide; FVC: forced vital capacity; ILD: interstitial lung disease; IQR: interquartile range; PFT: pulmonary function test; RP-ILD: rapidly progressive ILD. * Deterioration of PFT is defined as a decline in FVC ≥ 10% and DLCO ≥ 15%. ** Hypoxemia is defined as oxygen saturation level (SaO_2_) < 95% or arterial partial pressure of oxygen (PaO_2_) < 60 mmHg. ^&^ per 100 PY (patient-year).

## Data Availability

Deidentified participant data will be available from authors after publication through mariadelrosario.garcia@salud.madrid.org. Qualified researchers whose proposed use of the data has been approved for specified purpose can access the data after approval of a proposal.
